# Activation of Spinal **α**2-Adrenoceptors Using Diluted Bee Venom Stimulation Reduces Cold Allodynia in Neuropathic Pain Rats

**DOI:** 10.1155/2012/784713

**Published:** 2012-08-27

**Authors:** Suk-Yun Kang, Dae-Hyun Roh, Ji-Ho Park, Hye-Jung Lee, Jang-Hern Lee

**Affiliations:** ^1^Department of Veterinary Physiology, College of Veterinary Medicine and Research Institute for Veterinary Science, Seoul National University, Seoul 151-742, Republic of Korea; ^2^Department of Maxillofacial Tissue Regeneration, School of Dentistry, Kyung Hee University, Seoul 130-701, Republic of Korea; ^3^Graduate School of East-West Medical Science, Kyung Hee University, Kyunggi-do 446-701, Republic of Korea; ^4^Acupuncture and Meridian Science Research Center, Kyung Hee University, Seoul 130-701, Republic of Korea

## Abstract

Cold allodynia is an important distinctive feature of neuropathic pain. The present study examined whether single or repetitive treatment of diluted bee venom (DBV) reduced cold allodynia in sciatic nerve chronic constriction injury (CCI) rats and whether these effects were mediated by spinal adrenergic receptors. Single injection of DBV (0.25 or 2.5 mg/kg) was performed into Zusanli acupoint 2 weeks post CCI, and repetitive DBV (0.25 mg/kg) was injected for 2 weeks beginning on day 15 after CCI surgery. Single treatment of DBV at a low dose (0.25 mg/kg) did not produce any anticold allodynic effect, while a high dose of DBV (2.5 mg/kg) significantly reduced cold allodynia. Moreover, this effect of high-dose DBV was completely blocked by intrathecal pretreatment of idazoxan (**α**2-adrenoceptor antagonist), but not prazosin (**α**1-adrenoceptor antagonist) or propranolol (nonselective **β**-adrenoceptor antagonist). In addition, coadministration of low-dose DBV (0.25 mg/kg) and intrathecal clonidine (**α**2-adrenoceptor agonist) synergically reduced cold allodynia. On the other hand, repetitive treatments of low-dose DBV showing no motor deficit remarkably suppressed cold allodynia from 7 days after DBV treatment. This effect was also reversed by intrathecal idazoxan injection. These findings demonstrated that single or repetitive stimulation of DBV could alleviate CCI-induced cold allodynia via activation of spinal **α**2-adrenoceptor.

## 1. Introduction

Neuropathic pain, which is related to the development of hyperalgesia (an increased response to a stimulus that is normally painful) and allodynia (pain as a result of a stimulus that does not provoke pain), is one of several forms of chronic pain in humans and animals [1]. Although numerous studies on neuropathic pain treatment have attempted to elucidate pathophysiological mechanisms in humans and experimental animals, the presence of several side effects, such as motor dysfunction, sedation, dependence, and tolerance, have limited their application for the treatment of this chronic pain condition [2, 3]. In particular, a complaint of neuropathic pain patients is an evoked pain sensation to innocuous cold stimuli, that is, cold allodynia [4], which has been also studied in several neuropathic animal models [5, 6]. Cold allodynia in neuropathic pain has been reported to be associated with various pathophysiologic changes including peripheral and central sensitization [7]. Despite the importance of cold allodynia in neuropathic pain patients, the distinct effects of various treatments are still unclear.

Chemical acupuncture point (acupoint) stimulation with diluted bee venom (DBV), termed *apipuncture*, has been used clinically in traditional oriental medicine to produce a potent antinociceptive effect in human patients [8]. Previous experimental studies in our laboratory provide support for this alternative medicine approach by demonstrating that a prominent antinociceptive and antihyperalgesic effect is produced by apipuncture in several animal models of pain including the formalin test, the writhing test, the carrageenan-induced inflammatory pain test, and models of arthritis [9–12]. 

Our recent study has also shown that a single injection of DBV into Zusanli acupoint temporarily alleviated only thermal hyperalgesia, but not mechanical allodynia in the rat sciatic nerve chronic constrictive injury (CCI) model of neuropathic pain [13]. In addition, repetitive DBV treatment for 2 weeks produced a significant analgesic effect on both thermal hyperalgesia and mechanical allodynia in the rat sciatic nerve CCI model [14]. Furthermore, the intrathecal clonidine-induced analgesia was significantly enhanced when it was combined with chemical acupuncture treatment, and administration of low-dose clonidine in combination with acupuncture produced a potent analgesic effect without significant side effects in the CCI model [15]. We have further shown that this DBV-induced analgesic effect is mediated by the activation of descending coeruleospinal noradrenergic pathways, which in turn activate spinal *α*2 adrenoceptors [10, 15, 16]. Despite the antinociceptive effectiveness of chemical acupuncture using DBV, no data are available on the potential effect and underlying mechanism of single or repetitive DBV on suppression of cold allodynia accompanying peripheral nerve injury.

Therefore, we examined whether single or repetitive DBV treatment reduces cold allodynia in CCI rats and whether these effects of DBV are mediated by spinal adrenergic receptor activation. In addition, we verified whether single or repetitive DBV affects motor function in CCI rats using the accelerating rotarod test. 

## 2. Materials and Methods

### 2.1. Experimental Animals

Experiments were performed on male Sprague-Dawley rats weighing 180–230 g. All experimental animals were obtained from the Laboratory Animal Center of Seoul National University. They were housed in colony cages with free access to food and water and maintained in temperature- and light-controlled rooms (24 ± 2°C, 12/12 h light/dark cycle with lights on at 07 : 00). All of the methods used in the present study were approved by the Animal Care and Use Committee at Seoul National University and conformed to the NIH guidelines (NIH publication no. 86-23, revised 1985). 

### 2.2. Neuropathic Surgery

The CCI surgery of the common sciatic nerve was performed according to the method described by Bennett and Xie [17]. Briefly, rats were anesthetized with 3% isoflurane in a mixture of N_2_O/O_2_ gas. The left sciatic nerve was exposed at the midthigh level, and 4 loose ligatures of 4-0 chromic gut were placed around the dissected nerve with a 1.0 to 1.5 mm interval between each ligature. Sham surgery consisted of exposing the sciatic nerve in the same manner, but without ligating the nerve. During recovery, animals were housed in clear plastic cages with a thick layer of sawdust bedding.

### 2.3. Experimental Groups and Treatment

First, animals were divided into four groups to test the effect of single DBV treatment: [1] sham surgery animals that did not receive any treatment (Sham: Naive, *n* = 6), [2] CCI surgery animals that received a single saline injection (CCI: Sal, *n* = 6), [3] CCI surgery animals that received a single DBV (2.5 mg/kg) injection (CCI: DBV (2.5 mg), *n* = 6), and [4] CCI surgery animals that received a single DBV (0.25 mg/kg) injection (CCI: DBV (0.25 mg), *n* = 6). In addition, to confirm the effect of repetitive treatment of saline and DBV, two groups were added [5]: CCI surgery animals that received repetitive saline injections (CCI: R-Sal, *n* = 8) and [6] CCI surgery animals that received repetitive DBV injections (CCI: R-DBV (0.25 mg), *n* = 8). DBV (Sigma, St. Louis, MO) was first dissolved in a 50 *μ*L volume of saline and the DBV solution was subcutaneously injected into the Zusanli acupoint (ST36) on the same side as the CCI surgery (ipsilateral side). The Zusanli point was located 5 mm below and lateral to the anterior tubercle of the tibia. Animals in groups 2, 3, and 4 received a single injection of saline or DBV, respectively, at 2 weeks after CCI surgery. Repetitive DBV or saline injections started at day 15 after CCI surgery and administered twice a day (at 8 AM and 8 PM, resp.) for 2 consecutive weeks.

In order to verify the effects of DBV on cold allodynia and its mechanisms related to the spinal adrenergic systems in CCI surgery animals, we used prazosin (PRA, *α*1 adrenoceptor antagonist, 50 *μ*g), idazoxan (IDX, *α*2 adrenoceptor antagonist, 50 *μ*g), and propranolol (PRO, nonselective *β* adrenoceptor antagonist, 50 *μ*g). All drugs were administered by intrathecal injection, based on the technique developed by Hylden and Wilcox [18]. Briefly, animals were weakly anesthetized with 3% isoflurane in a mixture of N_2_O/O_2_ gas and a 26-gauge needle (length, 1 inch) connected to a 25 *μ*L Hamilton syringe which was inserted into the subarachnoidal space between the lumbar vertebrae L5 and L6. A flick of the rat's tail was a reliable indicator that the needle had penetrated the dura. The syringe was held in position for a few seconds after the injection of a volume of 10 *μ*L/rat. All drugs were purchased from Sigma (St. Louis, MO) and dissolved in sterile 0.9% NaCl (normal saline). These drugs were injected for 5 min before single DBV or saline administration into the Zusanli acupoint. In repetitive experimental groups, IDX was injected 4 hours after the last DBV and saline treatment. 

Finally, to assess the effect of combined DBV and clonidine (CLO, *α*2 adrenoceptor agonist, 1, 5, and 15 nmol), CLO was injected 5 min after DBV injection, and the control groups consisted of animals that were injected with vehicle instead of CLO and DBV.

### 2.4. Behavior Assessment for Cold Allodynia

**Figure 1:**
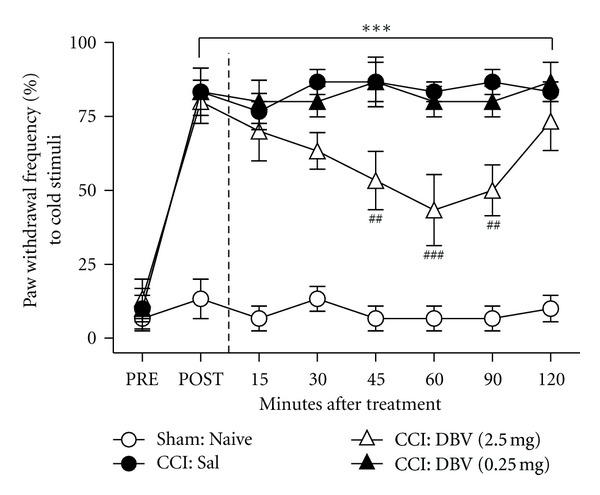
Effect of single DBV treatment on cold allodynia. A graph illustrating the effect of single treatment with diluted bee venom (DBV) on cold allodynia in neuropathic rats. All the CCI-subjected animal groups (Saline, DBV (2.5 mg), DBV (0.25 mg)) significantly increased paw withdrawal frequency (PWF, %) to innocuous cold stimulus compared with that of the sham surgery animals (Sham, ***P < 0.001). Moreover, the high-dose DBV treatment group (DBV (2.5 mg)) significantly suppressed the CCI-induced PWF from 45 minutes to 90 minutes after DBV treatment compared with the saline-treated group (Saline, ^##^ *P* < 0.01 and ^###^ *P* < 0.001).

Behavior assessments were performed 1 day prior to the CCI surgery to obtain normal baseline values of withdrawal responses to cold stimuli (PRE abbreviated in Figure 1). Fourteen days after surgery, all experimental animals were behaviorally tested as described below to confirm the development of neuropathic pain (POST abbreviated in Figure 1). Animals were randomly assigned to individual treatment groups and all subsequent behavioral tests were performed blindly. During the experiment, behavioral testing was performed at the same time each day (from 2 PM to 6 PM) at a constant room temperature (25 ± 1°C) to avoid any diurnal variation. 

Cold allodynia was measured as the presence of foot withdrawal responses (lifting, shaking, or licking) after the application of cold stimuli to the plantar surface of the paw [19, 20]. A drop of 100% acetone was gently applied to the operated paw of the rat with a 1 cc syringe connected to a PE10 tube. The test was repeated five times with an interval of approximately three to 5 min between each test. The response frequency to acetone was expressed as a percent response frequency. 

### 2.5. Accelerating Rotarod Test

The rotarod test is typically used to examine possible deficits in motor function including motor incoordination and ataxia in rodents [21]. Before and after the CCI surgery, forced motor performance was tested using a standard rotarod apparatus (Dae-Jong Engineering & Clean Technology, Korea Model no. DJ-4009). Animals were placed on a rotating rod (12 cm wide; 6 cm diameter) suspended 33 cm above the bottom of the apparatus. Escape to either side was prevented by plexiglas walls. The accelerating rotarod was set to accelerate gradually from a speed of 4 to 40 rpm for each trial. The starting speed was 4 rpm, and the total time of each trial was 300 sec or until the animal fell from the platform. On a given trial, four rats were placed on the cylinder, one in each compartment. Rats were tested three times in each testing time, with a minimum of 5 min of rest in the home cage between trials. Animals were tested before and at 1 and 2 hours after DBV injection.

### 2.6. Statistical Analysis

All values are expressed as mean ± SEM. Statistical analysis was performed using Prism 5.0 (Graph Pad Software, San Diego, CA, USA). Repeated measures two-way ANOVA was performed to determine the overall differences at each time point in PWF (%) and accelerating rotarod performance. Post hoc analysis was performed using the Bonferroni's multiple comparison test in order to determine the *P* value among the experiment groups. An unpaired Student's *t*-test was also used to analyze the effects of repeated idazoxan in the DBV-repeated treatment animals. A *P* < 0.05 was considered statistically significant. 

## 3. Results

### 3.1. Effect of Single DBV Treatment on CCI-Induced Cold Allodynia

The paw withdrawal frequency (PWF, %) to cold stimulus (a drop of 100% acetone) was significantly increased in all groups subjected to CCI surgery (CCI: Sal, DBV (2.5 mg), DBV (0.25 mg)) compared with that of the sham surgery animals (Sham: Naive, ***P < 0.001) as shown in Figure 1. The single-high-dose DBV group (CCI: DBV (2.5 mg)) injection at 2 weeks after CCI significantly reversed the CCI-induced PWF increase from 45 min to 90 min after DBV treatment compared with the saline-treated group (CCI: Sal, ^##^ *P* < 0.01 and ^###^ *P* < 0.001, Figure 1). However, the animals of single-low-dose (CCI: DBV (0.25 mg)) DBV injection did not have an effect at any time point.

### 3.2. Effect of Intrathecal Adrenoceptor Antagonists on Single DBV-Induced Anticold Allodynic Effect

**Figure 2:**
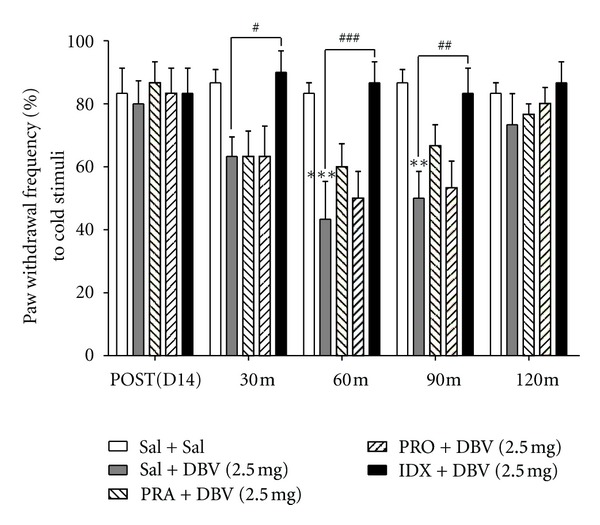
Effect of intrathecal adrenoceptor antagonist injection on the DBV-induced antinociception. The effects of intrathecal adrenoceptor antagonists on the DBV-induced antiallodynic effect to cold stimuli in neuropathic pain animals. The saline pretreatment in DBV-treated rats (Sal + DBV) reduced paw withdrawal frequency (PWF, %) to cold stimulus at 60 and 90 minutes compared with the DBV-treated group (Sal + Sal, **P < 0.01 and ***P < 0.001). The idazoxan-pretreated group (IDX + DBV), but not the PRA- and PRO-treated groups (PRA + DBV and PRO + DBV), significantly reversed PWF from 30 minutes to 90 minutes compared with the saline pretreatment group of DBV-treated rats (Sal + DBV, ^#^ *P* < 0.05, ^##^ *P* < 0.01 and ^###^ *P* < 0.001). All groups were comprised of six animals, respectively. PRA: prazosin, *α*1-adrenoceptor antagonist, IDX: idazoxzn, *α*2-adrenoceptor antagonist, PRO: propranolol, nonselective *β*-adrenoceptor antagonist.

The effect of intrathecal adrenoceptor antagonists (PRA: *α*1-adrenoceptor antagonist, IDX: *α*2-adrenoceptor antagonist, PRO: nonselective *β*-adrenoceptor antagonist) pretreatment after DBV (2.5 mg) treatment is illustrated in Figure 2. Before the intrathecal adrenoceptor antagonists treatment experiment, we tested the effect of intrathecal saline pretreatment in saline- or DBV-treated rats (Sal + Sal and Sal + DBV (2.5 mg), resp., *n* = 6). Intrathecal saline pretreatment did not alter DBV's antinociceptive effect on cold allodynia. The PWF (%) in the DBV treatment group pretreated with intrathecal IDX (IDX + DBV (2.5 mg), *n* = 6) significantly reversed the decreased response to cold stimulus by DBV injection compared to the saline pretreatment group of DBV-treated rats (Sal + DBV (2.5 mg)) from 30 min to 90 min of IDX injection (^#^ *P* < 0.05, ^##^ *P* < 0.01, and ^###^ *P* < 0.001). On the other hand, the intrathecal PRA and the PRO-treated group (PRA + DBV (2.5 mg) and PRO + DBV (2.5 mg), resp., *n* = 6) did not produce any effect on cold allodynia during 120 min after BV treatment.

### 3.3. Effect of Combined DBV and Clonidine on CCI-Induced Cold Allodynia

**Figure 3:**
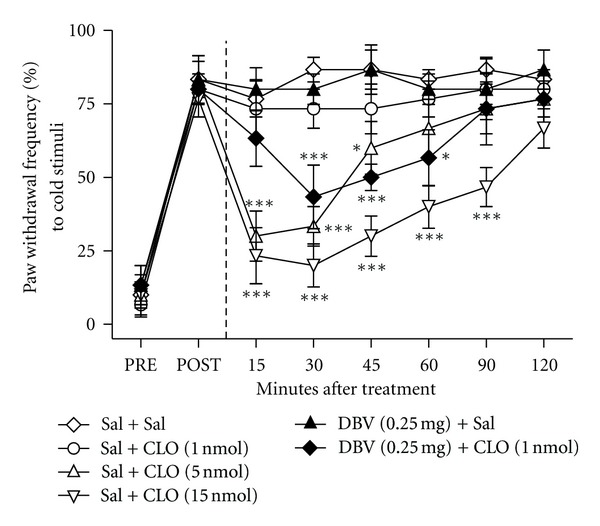
Effect of combined DBV and clonidine treatment on neuropathic pain-induced cold allodynia. A graph demonstrating the analgesic effects of intrathecal clonidine alone or in combination with injection of subcutaneous diluted bee venom (DBV) on CCI-induced cold allodynia. Rats were injected intrathecally with saline (Sal) or clonidine (CLO) 5 minutes after subcutaneous injection of saline or DBV. The intrathecal clonidine-injected rats (Sal + CLO (5 nmol) and Sal + CLO (15 nmol)) dose-dependently suppressed CCI-induced cold allodynic behavior compared with the saline-treated group (Sal + Sal, *P < 0.05 and ***P < 0.001) while the low-dose clonidine group (Sal + CLO (1 nmol)) and DBV injection without clonidine treatment (DBV (0.25 mg) + Sal) had no effect. The combined group of DBV acupuncture and low-dose clonidine (DBV (0.25 mg) + CLO (1 nmol)) significantly reversed CCI-injured cold allodynia compared with the control group (Sal + Sal, *P < 0.05 and ***P < 0.001).

This result shows the antiallodynic effect of intrathecal clonidine (CLO) alone and combination with DBV (0.25 mg/kg) injection into the Zusanli acupoint on PWF to cold stimuli. Intrathecal treatment with CLO suppressed cold allodynia in a dose-dependent manner. The highest dose (15 nmol) of CLO (Sal + CLO (15 nmol), *n* = 6) produced a strong antinociceptive effect on cold allodynia from 15 min to 90 min after treatment compared with the saline-treated group (Sal + Sal, *n* = 6, ***P < 0.001) and the moderate dose (5 nmol) of CLO (Sal + CLO (5 nmol) *n* = 6) significantly reversed the CCI-induced cold allodynia from 15 min to 45 min after CLO treatment compared with the saline-treated group (*P < 0.05 and ***P < 0.001), whereas the lowest dose (1 nmol) group (Sal + CLO (1 nmol) *n* = 6) tested in this study had no effect on CCI-induced cold allodynia, as shown in Figure 3. Although DBV injection into the Zusanli acupoint without CLO treatment (DBV (0.25 mg) + Sal, *n* = 6) did not produce antiallodynic effect and intrathecal injection of 1 nmol CLO also had no effect on CCI-induced cold allodynia, when intrathecal injection of 1 nmol CLO was paired with DBV apipuncture pretreatment (DBV (0.25 mg) + CLO (1 nmol), *n* = 6), it significantly reversed PWF at the 30, 45, and 60 min (after DBV injection) time points compared with the control group (subcutaneous saline + intrathecal saline, *P < 0.05 and ***P < 0.001).

### 3.4. Effect of Single or Repetitive DBV on Accelerating Rotarod Performance in CCI Rats

In order to determine whether DBV treatment might have a direct effect on motor activity in CCI animals, we used the accelerating rotarod test and measured duration of time that the animals spent on the rotarod. We found that the CCI surgery group that received a DBV (0.25 mg/kg) injection (CCI: DBV (0.25 mg), *n* = 6) did not produce any significant changes at any experimental time point after CCI surgery compared with that of the saline-treated animals (CCI: Saline, *n* = 6). However, the CCI surgery group that received single DBV (2.5 mg/kg) injection (CCI: DBV (2.5 mg), *n* = 6) showed a significant decreased duration of time on the rotarod at 60 min and 120 min after DBV treatment compared with the saline-treated animals (*P < 0.05 and **P < 0.01 in Table 1).

In addition, we have recently reported that the duration of time that the repetitive DBV treatment animals spent on the rotarod did not differ from the repetitive saline treatment group in the sham surgery animals [14]. The results from the present study also show that the repetitive DBV treatment in CCI animals (R-DBV: 0.25 mg) had no effect on duration of time spent on the rotarod after daily treatments (measured on days 4–13) compared with the repetitive saline-treated CCI rats (R-Saline, Table 2). 

### 3.5. Effect of Repetitive DBV Treatment on CCI-Induced Cold Allodynia

**Figure 4:**
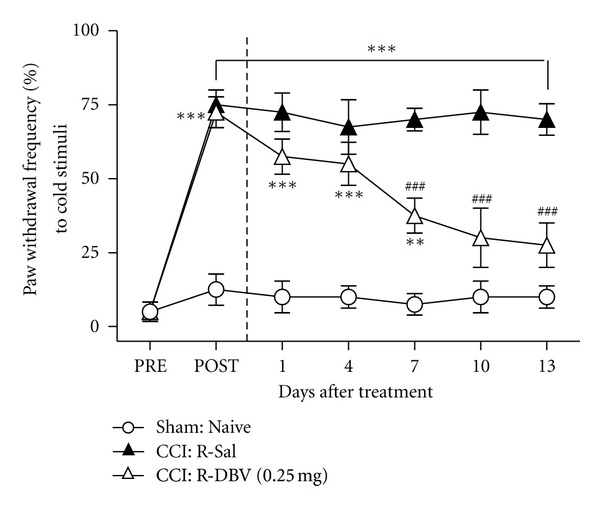
Effect of repetitive DBV treatment on cold allodynia. A graph illustrating the effect of repetitive treatment with diluted bee venom (DBV) from 2 weeks after CCI on cold allodynia in neuropathic rats. The repetitive saline injection group (CCI: R-Sal) significantly increased the paw withdrawal frequency (PWF, %) to 13 days after repetitive saline injection (***P < 0.001 compared with the sham surgery animals, Sham: Naive). The repeated DBV-treated animals (CCI: R-DBV (0.25 mg)) also increased the PWF to day 7 compared with the sham surgery group (**P < 0.01 and ***P < 0.001) and reversed the PWF from 7 days compared with the saline-treated group animals (^###^ *P* < 0.001).

In order to determine if repetitive low-dose (0.25 mg/kg) DBV, having no effect with single injection, might have an anticold allodynic effect in CCI animals, DBV and saline injections were started at day 15 after CCI surgery for 2 consecutive weeks. The PWF to cold stimulus of the repetitive saline injection group (CCI: R-Sal) was increased (approximately 75%) from 2 weeks after CCI surgery (POST) until day 13 after repetitive saline injection (***P < 0.001 compared with the sham surgery animals, Sham: Naive). Repeated administration of BV (CCI: R-DBV (0.25 mg)) significantly increased the PWF from day 1 to day 7 compared with the sham surgery group (**P < 0.01 and ***P < 0.001). In particular, repeated administration of BV (CCI: R-DBV (0.25 mg)) significantly reduced the PWF from 7 days compared with the saline-treated group animals (CCI: R-Sal, ^###^ *P* < 0.001) in Figure 4.

### 3.6. Effect of Intrathecal *α*2-Adrenoceptor Antagonist on Repetitive DBV-Induced Anticold Allodynic Effect

**Figure 5:**
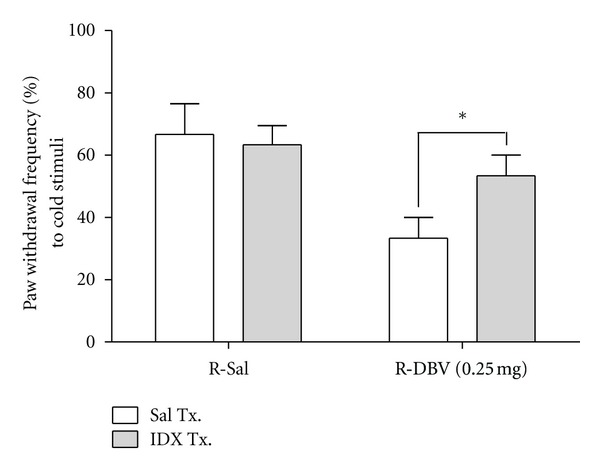
Effect of intrathecal *α*2-adrenoceptor antagonist injection on the repetitive DBV-induced antinociception. The effects of intrathecal *α*2-adrenoceptor antagonists on repetitive diluted-bee-venom-(DBV-) induced decrease of cold allodynia in CCI animals. Saline and idazoxan were intrathecally injected after repetitive saline and DBV treatment for 2 consecutive weeks. In the repetitive saline injection group, intrathecal idazoxan injection (R-Sal: Idazoxan) did not change the cold allodynic behavior compared with the saline-injected animals (R-Sal: Saline), whereas intrathecal idazoxan injection in the repetitive DBV group (R-DBV (0.25 mg): Idazoxan) significantly reversed the reduced response to cold stimulus compared with the saline-treated rats (R-DBV (0.25 mg): Saline, *P < 0.05).

In order to determine whether antiallodynic effect of repetitive low-dose (0.25 mg/kg) DBV also involves *α*2-adrenoceptor, similar with single DBV injection, saline and IDX were injected after repetitive saline and DBV treatment for 2 consecutive weeks (at day 28 after CCI surgery). The intrathecal IDX-injected animals in the repetitive saline injection group (R-Sal: IDX, *n* = 6) had no effect on the PWF (%) to cold stimulus compared with the saline-injected animals (R-Sal: Sal, *n* = 6). In the repetitive DBV injection group, the intrathecal IDX-injected animals (R-DBV (0.25 mg): IDX, *n* = 6) significantly reversed the decreased response to cold stimulus by repetitive DBV treatment compared with the saline treatment rats (R-DBV (0.25 mg): Sal, *n* = 6, *P < 0.05) in Figure 5.

## 4. Discussion

The present study demonstrated that single (2.5 mg/kg) or repetitive DBV (0.25 mg/kg) produced the antiallodynic effect on cold allodynia in the sciatic nerve CCI-induced neuropathic animals. On the other hand, the treatment with single DBV injection with a low dose (0.25 mg/kg) did not reduce cold allodynia. Previous experimental studies in our laboratory have also demonstrated that a single injection of DBV (0.25 mg/kg) into the acupoint temporarily reduced thermal hyperalgesia (up to 45 min after DBV injection), but not mechanical allodynia in CCI rats [13]. However, repetitive DBV administration with the same dose for 2 weeks produced a gradual recovery of mechanical allodynia as well as thermal hyperalgesia in CCI-induced animals, and these analgesic effects were closely associated with an increase in noradrenergic neuronal activity in the LC with a significant suppression of pNR1 expression in the dorsal horn of the spinal cord [14]. Based on these findings, we hypothesized that repetitive DBV treatment with a low dose (0.25 mg/kg) was also able to produce the anticold allodynic effect in CCI animals and elucidated that that repetitive DBV treatment could be a gradual and significant therapeutic approach for cold allodynia in neuropathic pain. 

Recently, the transient receptor potential (TRP) channel family has been proposed to play an important role in thermosensation in mammals. TRPM8 and TRPA1 of many thermosensitive ion channels, including TRP channel, are responsive to cold stimuli [22]. TRPM8 is a ligand-gated nonselective cation channel involved in detection of sensations such as coolness. A recent study has reported that TRPM8-mediated cold sensitivity on nociceptive afferent neurons provides a mechanism of cold allodynia in rats with CCI [23]. An increase of TRPM8 RNA expression in sensory neurons is also associated with mechanical and cold hypersensitivity in neuropathic rats [24], and TRPM8-mediated analgesic effects are supported by a study using TRPM8 knockout mice [25]. These findings suggested that TRPM8 ion channel may be closely related to cold sensitivity induced by neuropathic animals. However, most studies are focused on peripheral signaling since these TRPM8 are mainly expressed in sensory neurons.

On the other hand, spinal cord *α*-adrenoceptors have been demonstrated to play an important role in the inter-spinal modulation of cold allodynia via the noradrenergic pain inhibitory system [26]. An intrathecal injection of *α*-adrenoceptor antagonist enhances pain-related responses [27], suggesting that the noradrenergic system has a tonic inhibitory influence on pain signaling in the spinal cord. In addition, several studies showed that systemic administrations of PRA (*α*1-adrenoceptor antagonist) dose-dependently attenuated cold allodynia, whereas yohimbine (*α*2-adrenoceptor antagonist) exacerbated the condition in a rat tail model of neuropathic pain [28]. Kim et al. also indicated that individual differences in the sensitivity of cold allodynia to phentolamine may be due to the difference in the *α*-adrenoreceptor subtype (i.e., *α*1- or *α*2-adrenoreceptor) predominantly involved in cold allodynia [29]. These results showed that both *α*1- and *α*2-adrenoceptors are involved in the adrenergic mechanism of cold allodynia in neuropathic pain.

In the present study, we investigated which spinal adrenergic receptors are related to the anticold allodynic effect of single DBV treatment with a high dose (2.5 mg/kg). The current experiment showed that intrathecal pretreatment with IDA (*α*2-adrenoceptor antagonists) but neither PRA (*α*1-adrenoceptor antagonist) nor PRO (nonselective *β*-adrenoceptor antagonist) was able to completely block the anticold allodynic effect of single DBV stimulation in the neuropathic pain model. Furthermore, intrathecal injection with IDA after repetitive low-dose DBV treatment for 2 weeks significantly reversed the reduction of withdrawal response to cold stimuli. These results indicated that the anticold allodynic effect of DBV treatment is mediated by spinal cord *α*2-adrenergic receptors. 

Previous studies from our laboratories with the acute pain model have suggested that the antinociceptive effect of DBV acupoint stimulation on acetic acid-induced writhing and formalin test was associated with activation of the descending noradrenergic system and specifically involved spinal cord *α*2-adrenoceptors [9, 30]. We have also reported that the DBV-induced antiallodynic and antihyperalgesic effect was blocked by intrathecal pretreatment with *α*2-adrenoceptor antagonists in the rodent neuropathic pain model [13, 15]. Therefore, our results indicate that the DBV-induced anticold allodynic response also mediates distinctive activation of the spinal cord *α*2 adrenergic system, but not the *α*1 and *β* adrenergic systems in the animal model of chronic neuropathic pain. 

We have previously demonstrated that DBV acupuncture alone or in combination with a low dose of intrathecal CLO produced an analgesic effect similar to that of a high dose of CLO in the formalin test, but without significant side effects [15]. In the present study, intrathecal CLO injection was shown to suppress peripheral nerve-injury-induced cold allodynic behavior in a dose-dependent manner, which is consistent with other previous studies [31, 32]. An important finding in the present study was that low-dose DBV acupuncture or CLO treatment alone did not induce any changes in pain behavior to cold stimuli, whereas combined treatment of DBV acupuncture and CLO with noneffective dose suppressed cold allodynia in CCI-injured rats. Therefore, our results indicate that DBV acupuncture is able to potentiate the CLO-induced suppressive effect on CCI-induced nociceptive activity.

Finally, we verified that a high dose of DBV (2.5 mg/kg) injected into an acupoint produced a deficit of motor function at 60 and 120 min and recovered at 240 min after single injection, while treatment with a low dose of DBV (0.25 mg/kg) did not affect motor function in the accelerating rotarod test (Table 1). We have recently found that the repetitive DBV (0.25 mg/kg) treated animals had no effect on duration of time spent on the rotarod in the sham surgery animals [14]. In this study, our results also showed that the repetitive DBV treatment in CCI-injured rats did not change the time spent on the rotarod after daily treatments compared with those in the saline-treated animals (Table 2). These results suggest that repetitive DBV therapy with low dose (0.25 mg/kg) for 2 weeks could be safe and provide potent anticold allodynic effects without motor impairment in neuropathic rats.

## 5. Conclusion

The current study showed that the single or repetitive treatment of DBV in acupoint potently suppressed cold allodynic response, and these anticold allodynic effects were completely reversed by spinal *α*2-adrenoceptor antagonist. In addition, coadministration of DBV treatment (low dose) and intrathecal CLO synergically reduced cold allodynia. Moreover, the repetitive treatment of low-dose DBV did not affect motor function in neuropathic rats. These findings demonstrated that DBV treatment could relieve cold allodynia via activation of spinal *α*2-adrenoceptors in neuropathic rats. In particular, repetitive DBV treatment at adequate dose may provide an improved strategy for cold allodynia management without motor dysfunction.

## Figures and Tables

**Table 1:** Effect of single DBV injection on accelerating rota-rod performance.

Group	Pre	Post	60 m	120 m	240 m
CCI (Saline)	100.24 ± 4.67	53.94 ± 5.33	52.51 ± 7.13	58.18 ± 8.68	56.25 ± 8.32
CCI (DBV: 2.5 mg)	103.14 ± 8.76	56.31 ± 5.36	31.91 ± 1.99**	36.49 ± 3.05*	51.30 ± 2.02
CCI (DBV: 0.25 mg)	116.68 ± 6.07	53.70 ± 4.23	51.29 ± 3.94	49.93 ± 5.17	60.28 ± 8.18

CCI (Saline): saline-treated CCI surgery animal group (*n* = 6), CCI (DBV: 2.5 mg): DBV 2.5 mg/kg treated CCI surgery animal group (*n* = 6), CCI (DBV: 0.25 mg): DBV 0.25 mg/kg treated CCI surgery animal group (*n* = 6) Pre: presurgery, Post: 2 weeks after CCI surgery, 60 m–240 m: minutes 60–240 after saline or DBV treatment.

**Table 2:** Effect of repetitive DBV injection on accelerating rota-rod performance.

Group	Pre	Post	D4	D7	D13
CCI (R-Saline)	95.10 ± 7.95	58.94 ± 6.44	64.10 ± 4.84	71.82 ± 5.67	78.42 ± 4.74
CCI (R-DBV: 0.25 mg)	100.40 ± 8.36	63.84 ± 8.44	64.04 ± 6.88	76.15 ± 3.15	87.20 ± 4.82

CCI (R-Saline): repetitive-saline-treated CCI surgery animal group (*n* = 6), CCI (R-DBV: 0.25 mg): repetitive DBV 0.25 mg/kg treated CCI surgery animal group (*n* = 6), Pre: pre-surgery, Post: 2 weeks after CCI surgery, D4, D7, and D13: days 4, 7, and 13 after initial saline or DBV treatment.
